# Management of high-risk (B3) breast lesions in symptomatic practice: evidence review and practical recommendations for multidisciplinary care

**DOI:** 10.1007/s00428-026-04672-2

**Published:** 2026-08-10

**Authors:** Emad A. Rakha, Abeer Shaaban, Giuseppe Floris, Grace Callagy, Nisha Sharma, Raghavan Vidya, Thomas Decker, Peter Regitnig, Yazan Masannat, Elena Provenzano, Rahul Deb, Jill R. Dietz, Basel Altrabulsi, William Teh, Muna A. Abuhejleh, Zsuzsanna Varga, Paul van Diest, Gábor Cserni, Maria Pia Foschini, Carolien H.M. van Deurzen, Madhuri Warren, Yasmeen Mir, Emmanuelle Charafe-Jauffret, Soha El Sheikh, Zsuzsanna Bago-Horvath, Isabella Castellano, Stephen Grobmyer, Mieke R. Van Bockstal, Puay Hoon Tan, Cecily Quinn, Sarah Pinder

**Affiliations:** 1 School of Medicine, University of Nottingham, Nottingham, UK; 2 Department of Pathology, National Reference Laboratory and Cleveland Clinic Abu Dhabi, Abu Dhabi, United Arab Emirates; 3 Cellular Pathology, Queen Elizabeth Hospital Birmingham, Birmingham, UK; 4 Department of Cancer and Genomic Sciences, University of Birmingham, Birmingham, UK; 5 Department of Pathology, University Hospitals Leuven, KU-Leuven, Herbstreit 49, Leuven, 3000 Belgium; 6 Discipline of Pathology, University of Galway, Galway, Ireland; 7 Breast Radiologist, Breast Unit, University of Galway, Galway, Ireland; 8 Oncoplastic Breast Surgery Department, The Royal Wolverhampton NHS Trust, Wolverhampton, UK; 9 Reference Centers Mammography, Münster and Berlin, Berlin, Germany; 10 Diagnostic and Research Institute of Pathology, Medical University of Graz, Graz, Austria; 11 Oncoplastic Breast Surgery Department, Broomfield Hospital, Mid and South Essex NHS Trust, Chelmsford, England; 12 Department of Histopathology, Addenbrookes Hospital, Cambridge, UK; 13 Department of Pathology, University Hospitals of Derby and Burton, Derby, UK; 14 Department of Surgery, NYULangone Health Medical Center, New York, NY USA; 15 Pathology and Laboratory Medicine Institute, Cleveland Clinic Abu Dhabi, Abu Dhabi, United Arab Emirates; 16 Radiology Department, Northwick Park Hospital & Royal Free Breast Screening, Harrow, Middlesex UK; 17 Department of Pathology and Molecular Pathology, University Hospital Zurich, Zurich, Switzerland; 18 Department of Pathology, University Medical Center Utrecht, Utrecht, The Netherlands; 19 Department of Pathology, Albert Szent-Györgyi Faculty of Medicine, University of Szeged, Bács-Kiskun County Teaching Hospital, Szeged, Kecskemét, Hungary; 20 Department of Biomedical and Neuromotor Sciences, Unit of Pathology at Bellaria Hospital, University of Bologna, Bologna, 40139 Italy; 21 Department of Pathology, Erasmus MC, Rotterdam, The Netherlands; 22 Department of Cellular Pathology, Barts Health NHS Trust, Queen Mary University of London, London, UK; 23 Department of Pathology, Liverpool University Hospitals Group, Liverpool, UK; 24 Pathology Department & Epithelial Stem Cells and Cancer Lab, CRCM, Inserm, CNRS, Institut Paoli-Calmettes, Aix-Marseille Univ, Equipe labellisée LIGUE contre le cancer, Marseille, France; 25 Research Department of Pathology, University College London, London, UK; 26 Department of Pathology, Medical University of Vienna, Vienna, Austria; 27 Department of Medical Sciences, University of Turin, Turin, Italy; 28 Surgical Department Breast Oncoplastic Unit, Cleveland Clinic Abu Dhabi, Abu Dhabi, United Arab Emirates; 29 Department of Pathology, UCLouvain, Cliniques universitaires Saint-Luc, Brussels, Belgium; 30 Luma Medical Centre, Royal Square Medical Centre, Singapore, Singapore; 31 Department of Histopathology, School of Medicine, Irish National Breast Screening Programme, St. Vincent’s University Hospital, University College Dublin, Dublin, Ireland; 32 School of Cancer and Pharmaceutical Sciences, King’s College London, London, UK

**Keywords:** B3 breast lesions, Symptomatic breast lesion management, Radiological-pathological correlation, Multidisciplinary approach, Vacuum-assisted biopsy, Vacuum-assisted excision

## Abstract

Borderline breast lesions (B3 lesions, also termed lesions of uncertain malignant potential or high-risk lesions) represent a heterogeneous group of entities associated with variable risks of malignancy. While the management of screen-detected B3 lesions has become increasingly standardised, no dedicated international recommendations exist for symptomatic B3 lesions, despite them posing a distinct clinical challenge. Symptomatic lesions differ from screen-detected lesions in their mode of presentation, lesion characteristics, biopsy techniques, and diagnostic objectives; consequently, management strategies derived from screening populations may not be directly applicable. This review summarises the current evidence and proposes a pragmatic management framework for B3 lesions encountered in the symptomatic setting (defined as those presenting with breast symptoms outside population-based screening programmes). This is particularly relevant for patients under 50 years of age, where the primary objective is to exclude malignancy at the index site rather than solely to stratify long-term cancer risk. Clinical-radiological-pathological concordance and multidisciplinary assessment are central to management decisions. Lesions presenting as palpable abnormalities (which are typically larger) or those demonstrating radiological-pathological discordance warrant a lower threshold for additional sampling or excision. Conversely, selected concordant lesions without atypia may be managed conservatively following adequate sampling. The long-term cancer risk associated with epithelial atypia should also be considered with subsequent risk-based surveillance implemented where appropriate. Overall, management requires a risk-adapted, multidisciplinary approach integrating pathological, radiological, and clinical factors alongside patient symptoms and quality-of-life considerations. Given the limited evidence, current recommendations rely on expert consensus and a proposed practical framework. They underscore the need for large-scale, high-quality studies with long-term follow-up to establish formal clinical guidelines.

**Supplementary information:**

The online version contains supplementary material available at https://doi.org/10.1007/s00428-026-04672-2.

## Introduction

A key limitation of percutaneous breast biopsy, including core needle biopsy (CNB) and vacuum-assisted biopsy (VAB), is potential under-sampling, whereby a malignant component is missed, and only associated benign changes are sampled, resulting in a benign but ‘of uncertain malignant potential’ (B3) diagnosis [1–7]. Although B3 is primarily a coding convention, these lesions are widely termed “high-risk lesions,” “borderline lesions,” or “lesions of uncertain malignant potential.” However, increased subsequent breast cancer risk is mainly confined to lesions with epithelial atypia, whereas several other B3 entities are associated with a very low concurrent carcinoma risk [7–10]. B3 lesions constitute a heterogeneous group and can be clustered into four broad categories: (1) epithelial atypia, including atypical intraductal epithelial proliferation (AIDEP)/atypical ductal hyperplasia (ADH), lobular neoplasia (LN; atypical lobular hyperplasia (ALH) and classical lobular carcinoma in situ (LCIS), flat epithelial atypia (FEA), and apocrine atypia; (2) benign lesions that may harbour atypia or malignancy, such as papilloma and radial scar/complex sclerosing lesion (RS/CSL); (3) heterogeneous lesions, particularly cellular fibroepithelial lesions (FELs), which often require complete excision for definitive classification; and (4) miscellaneous lesions, including low-grade spindle cell lesions, adenomyoepithelioma, vascular lesions, granular cell tumours and others [11–13].

B3 lesions are most commonly encountered in population-based breast cancer screening, where concerns regarding both underestimation of malignancy and overtreatment of asymptomatic individuals drive management. Large retrospective and prospective studies have informed increasingly standardised screening pathways incorporating VAB, vacuum-assisted excision (VAE), selective surgery, and surveillance [10, 14–20]. However, the applicability of these pathways to symptomatic practice remains uncertain. Management in symptomatic patients is inherently more complex owing to greater heterogeneity in clinical presentation, a more limited evidence base, and the need to interpret pathological findings within the broader clinical (and radiological) context. Additional considerations include younger patient age, cumulative lifetime breast cancer risk, patient preference, and the absence of well-defined surveillance protocols. Follow-up imaging may also require individualisation, as magnetic resonance imaging (MRI) or contrast-enhanced mammography (CEM) may offer advantages over conventional mammography in selected younger women [21, 22].

Historical surveillance strategies for screen-detected B3 lesions with atypia were largely precautionary and based on evidence generated before the widespread adoption of VAB. Contemporary image-guided vacuum-assisted management has enabled a more selective and risk-adapted approach to follow-up. Data from the Sloane project suggest that annual mammographic surveillance may not be necessary for all patients with screen-detected atypia after adequate assessment and management, highlighting the need for more tailored follow-up strategies [23, 24]. However, the applicability of these findings to symptomatic practice remains uncertain [25].

Symptomatic lesions account for only 20–40% of B3 diagnoses, and epithelial atypia is proportionally less common than in screening populations, thereby limiting the available evidence base [26–30]. For example, a large single-centre series of 4,168 biopsies reported B3 lesions in 6.5% of symptomatic cases compared with 12.4% of screening cases, with atypia present in 30.2% and 56.4% of lesions, respectively [28]. Consequently, current management recommendations are largely derived from screening cohorts, although this distinction is often not explicitly stated, and no dedicated international guidelines address the management of symptomatic B3 lesions [10, 15, 16].

**Fig. 1:**
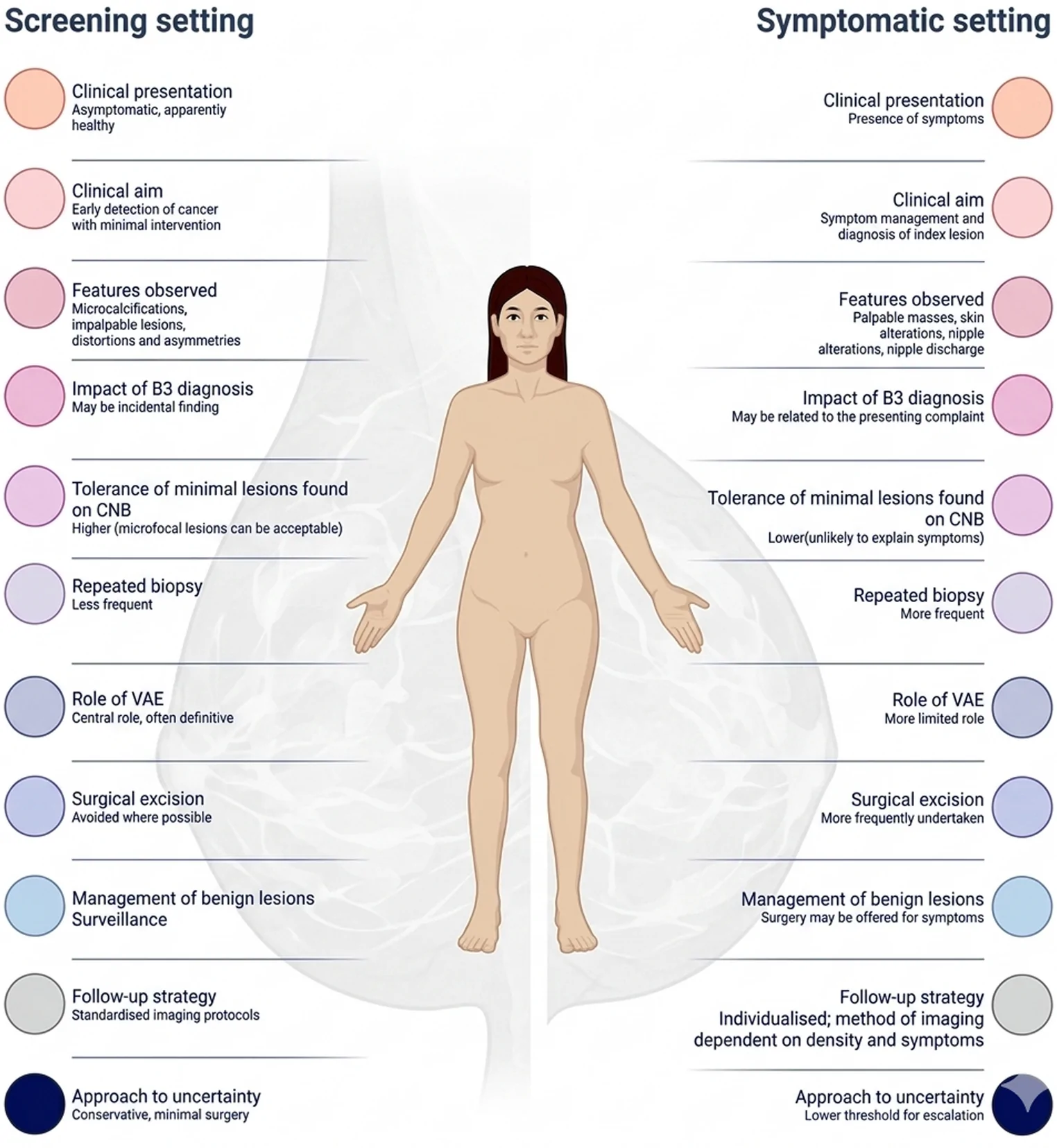
Comparison of B3 lesion management in screening and symptomatic settings. Schematic overview highlighting differences in the clinical context, diagnostic approach, and management of B3 breast lesions detected through population-based screening versus those presenting with symptoms. Figure created using BioRender.com

This review addresses the diagnostic, clinical, and management challenges of B3 lesions in symptomatic settings, including patients presenting outside structured screening programmes with symptoms (e.g. palpable mass, nipple discharge, pain, skin change) or undergoing opportunistic imaging based on clinical concern or perceived risk [31, 32]. The key differences between these settings are summarised in Table 1; Fig. 1. Given limited high-level evidence, recommendations are presented as an evidence-informed practical framework rather than as a formal guideline. Management of patients with equivocal C3 cytology associated with symptomatic B3 lesions is outside the scope of this review [33].

**Table 1:** Comparison of the clinical context and management principles for B3 breast lesions in screening and symptomatic practice

Feature	Screening setting	Symptomatic setting
Patient population	Asymptomatic individuals undergoing population-based screening	Patients presenting with breast symptoms or clinical abnormalities
Primary clinical objective	Early cancer detection while minimising unnecessary intervention	Identification of the cause of symptoms and diagnosis of the index lesion
Typical presentation	Microcalcifications, architectural distortion, asymmetries and small impalpable lesions	Palpable masses, nipple discharge, skin or nipple changes, imaging abnormalities associated with symptoms
Clinical significance of a B3 diagnosis	Frequently an incidental imaging finding	More likely to represent or be related to the presenting clinical abnormality
Clinicopathological-radiological concordance	Usually based on imaging findings alone	Requires close integration of clinical examination, imaging and pathology to ensure the biopsy adequately explains the presenting symptoms
Tolerance of minimal lesions on CNB	Higher; small incidental lesions may adequately explain imaging findings	Lower; minimal lesions may not account for symptoms, increasing concern for sampling error
Need for repeat biopsy	Less common	More frequent when clinicopathological-radiological discordance or inadequate sampling is suspected
Role of vacuum-assisted excision (VAE)	Central role; often provides definitive diagnosis and treatment	More selective role; often reserved for carefully selected concordant lesions
Role of surgical excision	Avoided where safe and supported by evidence	Lower threshold for surgical excision, particularly when symptoms persist or diagnostic uncertainty remains
Management of benign concordant lesions	Imaging surveillance is often appropriate	Surgery or additional intervention may be considered to alleviate symptoms despite benign pathology
Follow-up strategy	Standardised imaging surveillance according to screening protocols	Individualised follow-up based on symptoms, breast density, imaging findings and multidisciplinary assessment
Approach to diagnostic uncertainty	Conservative management with emphasis on avoiding overtreatment	Lower threshold for additional sampling, specialist pathological review and surgical management when uncertainty persists

*CNB* core needle biopsy

## Clinical context: Screening versus symptomatic practice

Population-based screening programmes assess asymptomatic individuals to maximise early cancer detection while minimising unnecessary intervention. Most B3 lesions are asymptomatic and detected through screening as microcalcifications or impalpable imaging abnormalities. Management focuses on securing a definitive diagnosis while avoiding overtreatment and false negative diagnoses. In contrast, symptomatic practice involves a broader and more heterogeneous population, often including younger patients. Presentations include palpable masses, breast pain, nipple discharge, or skin changes. Small focal lesions or isolated microcalcifications, common in screening populations, may instead represent incidental findings on opportunistic imaging undertaken on women with symptomatic lesions in the breast. This group may also include patients assessed outside routine screening, such as those with a known high-risk genetic mutation, a family history of breast cancer or previous breast cancer, those undergoing imaging investigation in the setting of contemporaneous ipsilateral or contralateral breast cancer, and individuals presenting with a symptom between screening rounds [34, 35]. High-risk and interval presentations overlap with symptomatic workflows but are not identical and are discussed separately; protocols for very high-risk family history patients are already in place [36, 37]. Accordingly, diagnostic uncertainty in these non-screening settings may have different clinical implications and often justifies a lower threshold for further investigation. As a result, diagnostic pathways, follow-up strategies, and multidisciplinary management may differ from lesions detected through routine breast screening practice.

### Approach to diagnosis

In current practice, image-guided (typically ultrasound-guided) CNB is usually the first diagnostic step in evaluating a symptomatic lesion. Although symptomatic lesions are often larger and more accessible, initial sampling may not be representative and further imaging (repeat ultrasound, CEM, or MRI) and repeat biopsy (usually with VAB) may help to clarify the nature of the patient’s presenting complaint and reduce the risk of underestimation.

A distinction should be made between diagnostic sampling procedures (CNB, VAB) and excisional techniques, including VAE and open surgical excision (diagnostic or therapeutic). CNB and VAB are primarily designed to obtain tissue for diagnosis, although VAB may incidentally achieve complete removal of small lesions depending on lesion size and the extent of sampling. In contrast, VAE is performed with the intention of completely removing of selected small lesions and therefore serves both diagnostic and therapeutic purposes. Surgical excision may be undertaken for either diagnostic or therapeutic reasons, depending on the clinical context. However, neither VAE nor diagnostic surgical excision guarantees complete lesion removal, particularly when the extent of the lesion is uncertain or multifocal. The relative roles of CNB, VAB, VAE and surgical excision are summarised in Supplementary Table 1.

In the UK and many European centres, CNB of B3 lesions may be followed by VAB for additional sampling or by VAE or surgery, using a stepwise approach, for excision and further evaluation [15]. VAE is the preferred minimally invasive technique when technically feasible and desirable [31, 32]. VAE is also valuable in symptomatic practice, although its role may be limited by larger lesion size or lesion type (e.g. cellular fibroepithelial lesions). In patients presenting with a mass, nipple discharge, or skin changes, small histological foci (0.1–2 mm range), often referred to in practice as “microfocal” abnormalities (e.g. ADH, ALH, classical LCIS, FEA), are more likely to represent incidental or co-existent findings, or part of a partially sampled higher-risk lesion. Depending on lesion size, histological findings and clinical context, open surgical procedures may be more frequent than in screening practice. If malignancy is identified on excision, patients proceed along standard oncological pathways. Where a benign diagnosis is established, further management should be guided by clinical symptoms and signs, patient preference, the estimated risk of continued lesion growth or progression, and the potential impact on future interventions.

#### Multidisciplinary assessment

Given the complexity and heterogeneity of B3 lesions, multidisciplinary team (MDT) discussion is essential. Integration of pathological findings, imaging features, patient risk factors, symptoms, surgical and cosmetic considerations, comorbidities and patient preferences enables tailored management while balancing diagnostic uncertainty against the risks of under- or overtreatment. Shared decision-making remains central to this process. A B3 result in the presence of suspicious clinical (P4/P5) or imaging (BI-RADS 4/5) findings should raise concern in both settings and warrants further tissue sampling in some form for clarification. Moreover, given the well-recognised interobserver variability in the diagnosis of several B3 lesions [38–40], many laboratories routinely perform internal reviews or hold consensus discussions among breast pathologists for diagnostically challenging cases. Where uncertainty persists, particularly when the diagnosis may influence patient management, referral for specialist opinion should be considered as best practice. Such a review complements multidisciplinary assessment and may improve diagnostic consistency and patient care.

### Diagnostic uncertainty and upgrade risk in symptomatic b3 lesions

Symptomatic B3 lesions, particularly those presenting with a clinically significant abnormality, are more likely to harbour significant pathology than screen-detected lesions [41–46]. Microfocal atypical lesions such as ADH but also FEA, ALH and classical LCIS, when associated with a discrete mass, are usually part of a more complex lesion or an incidental finding adjacent to the symptomatic abnormality [47].

Although some studies have reported similar upgrade rates in symptomatic and screen-detected B3 lesions, published data remain heterogeneous and largely retrospective, with variability in lesion definitions, biopsy techniques, and case selection [14, 26, 27]. In a comparative study, *Elsheikh et al. * [28]. reported a higher positive predictive value (PPV) for B3 lesions with atypia in symptomatic patients (14%) than in screening patients (9.7%). The upgrade risk (i.e., identification of in situ or invasive carcinoma on subsequent sampling/excision) varies by lesion subtype (Table 2), which forms the basis for risk-adapted management strategies. Upgrade rates appear to increase with patient age, lesion size, mass-forming imaging features, biopsy method, and radiological–pathological discordance [18, 26, 27, 53]. Higher upgrade rates have also been reported following CNB compared with VAB, likely reflecting differences in sampling adequacy [8]. In patients with symptomatic B3 lesions, the risk of upgrade is also influenced by clinical presentation and baseline risk profile. Management decisions should be individualised and based on multidisciplinary assessment rather than uniform lesion-based algorithms alone. There remains no clear consensus regarding the indications for excision or the optimal extent of excision in many symptomatic B3 lesions [26, 54–57].

**Table 2:** Risk of upgrade of individual B3 lesions in the screen-detected settings [10, 14–16, 20, 23, 48–52] and proposed management strategy for symptomatic practice*

Lesion	% risk of upgrade	Risk of subsequent carcinoma	Recommendations for Screen-detected presentation	Suggested symptomatic management Strategy
ADH	22% (5–50%)	At 5-year: 4.8–6.6% At 10-year: 13.9%	° 1 st line management = VAE ° If rad-path discordance with only initial CNB OSE or VAE ° Discuss always in an MDT setting.	° Similar approach. ° After excision: risk-based management as per high-risk patient protocols
LN	27% (0–40%)	At 5-year: 5.4–8.6% At 10-year: 17%	° VAE if lesion is visible on imaging. ° If rad-path concordance with small (e.g., < 10 mm) adequately sampled lesion surveillance ° OSE discordance or presence of residual abnormality	° Same approach ° After excision: risk-based management as per high risk patient
ALH	12%	At 5-year: 9.7% At 10-year: 15.4%		
LCIS classical	22%	At 5-year: 9.3% At 10-year: 11.8%		
FEA	Varies according to whether associated atypical intraductal epithelial proliferation 11% (range 2–21%); or pure FEA: 5% (1–16%).	At 5-year: 3.2–3.8% At 10-year: 7.2%	° VAE is generally recommended to remove at least 90% of microcalcifications for thorough sampling. ° Surveillance may be considered for isolated concordant FEA without associated ADH. ° OSE if there is coexisting ADH or sampling is limited and there is rad–path discordance. ° Isolated FEA with almost complete removal and rad–path concordance no follow-up is required.	° Similar approach. ° The clinical significance of FEA in palpable breast masses such as papilloma or FELs remains uncertain and management in such cases should focus on the index mass lesion.
Multiple B3 lesions/mixed atypia (e.g. ADH + LN; atypia in FEL)		At 5-year: 7.8%	° Multiple B3 components increase uncertainty. ° OSE of the mass lesion with atypia is preferred but VAE is an option depending on variety of features.	° Surgery is generally more appropriate than VAE. ° If no upgrade and fully excised risk-based management may apply, dependent on histology.
Papillary Lesions	CNB: No atypia: 4–9%; with atypia: 20–36%. Lower rate when VAB		° VAE for IDP without concomitant ADH for diagnostic confirmation and potential therapeutic removal. ° If sampling is representative and rad–path concordance is achieved further excision may not be required. ° OSE for IDP with concomitant ADH because of the higher risk of associated carcinoma and the need to assess the extent (mm) of the atypia.	° Similar approach. ° May require OSE for control of symptoms (e.g. duct excision for nipple discharge)
Radial Scar/Complex sclerosing lesion	No atypia: 0–10%. With atypia: 2–36%. Lower rate when VAB		° VAE aiming for thorough sampling/complete removal of the imaging abnormality is recommended. ° OSE should be considered when the lesion is large, or atypia is present or rad–path discordance exists.	° Same approach ° After excision if atypia present: risk-based management as per high risk patient
Cellular FEL	Risk is phyllodes tumour (PT)	Typically represents benign PT low risk of recurrence as malignant PT	° OSE to distinguish cellular fibroadenoma from benign PT. ° VAE can be attempted for small FEL lesions without features suspicious of borderline or malignant PT. ° If there is a risk of borderline PT–> OSE with a margin. ° Excised Benign PT: no imaging follow-up is needed.	° Same approach
Adenomyoepithelioma (AME)	Benign: increased risk of LR compared to papilloma. Atypical AME: increased risk of LR and may develop lymph node metastasis	No data	° Classical AME–> OSE ° Atypical AME or histologically concerning features OSE with clear margins and MDT discussion. ° If completely excised and benign no imaging follow-up.	
Mucocele-like lesions (MLL)	Benign but hypocellular mucinous carcinoma or DCIS need to be excluded.		° VAE is preferred. ° If rad-path concordance is achieved and no atypia conservative management may be appropriate in selected cases. ° OSE if there is atypia or lesion is large and suspicious on imaging. ° MLL without atypia, adequately sampled and rad-path concordance no follow-up is needed.	° Similar approach. ° If NCB shows MLL and there is a palpable mass: VAE or OSE to exclude hypocellular mucinous carcinoma based on the size of the lesion and presence of any epithelial atypia.
Vascular Lesions			° Diagnostic surgical excision is typically advised but based on imaging/clinical and histological features and correlation. ° If benign no follow-up	
Fibromatosis	Locally infiltrative.	No cancer risk	° Watch and wait (soft tissue protocols) ° 1 st line OSE is not recommended unless patient choice for symptomatic cases.	
Myofibroblastoma and granular cell tumour	Benign lesion	No cancer risk	° OSE. ° If benign diagnosis confirmed on OSE No follow-up is required	

*OSE* open surgical excision, *rad–path* radiological–pathological concordance, *CNB* core needle biopsy, *VAB* vacuum-assisted biopsy, *VAE* vacuum-assisted excision, *FEL* fibroepithelial lesion, *PT* phyllodes tumour, *ADH* atypical ductal hyperplasia, *LN* lobular neoplasia, *ALH* atypical lobular hyperplasia, *LCIS* lobular carcinoma in situ, *FEA* flat epithelial atypia, *IDP* intraductal papilloma, *MLL* mucocele-like lesion, *AME* adenomyoepithelioma, *MDT* multidisciplinary team, *LR* local recurrence

* These are proposed management considerations, not formal guidelines, and management recommendations should be interpreted in the context of multidisciplinary assessment and individual patient factors and preference

#### Long-term risk of malignancy in epithelial atypia

The distinction between upgrade risk at presentation (contemporaneous malignancy detected on further sampling/excision) and long-term (later progression) breast cancer risk is central to the management of symptomatic B3 lesions. Epithelial atypia is a recognised marker of increased future breast cancer risk [23, 48, 58] and may be best regarded as an indicator of field-wide susceptibility rather than as an obligate short-term precursor, with subsequent cancers arising many years later and affecting either breast [23, 29, 30, 59]. Importantly, early cancer events after modern VAB/VAE assessment are uncommon, and rates appear comparable to those after surgical excision, supporting the safety of contemporary minimally invasive management in selected concordant cases [23].

Meta-analyses and cohort studies report an approximately fourfold increase in long-term breast cancer risk after a histological diagnosis of atypia, with 5- and 10-year incidence varying by lesion type and biopsy techniques [23, 48, 60, 61]. The number of atypical foci may also influence the cumulative risk of developing breast cancer [58]. A retrospective single institution review of 58 women under the age of 35, with histologically confirmed atypia (ADH, ALH, LCIS), reported a 12% rate of subsequent breast cancer with a median time to a diagnosis of malignancy of 90 months (range 37–231). Patients who developed malignancy included 6 with invasive carcinoma and 2 with DCIS. The authors concluded that, in view of the high subsequent breast cancer risk, young women should be followed up closely [62]. A large study of 530 patients investigated the effect of age (under 50, 50–70 and over 70) on long term outcome of atypical lesions [63]. The incidence of ADH, but not lobular neoplasia, steadily increased with age. Older age was associated with a significantly higher risk of contemporaneous malignancy but was not associated with an increased long-term risk of developing subsequent cancer. A recent systematic review of 12 studies (*n* = 6252 atypia) concluded that the currently available risk assessment models, including the Gail and Tyrer-Cuzick models, showed poor test concordance in risk prediction in patients with epithelial atypia. While the newer methods have improved calibration, further refinement and validation are required [64].

### Management principles in symptomatic practice

**Fig. 2:**
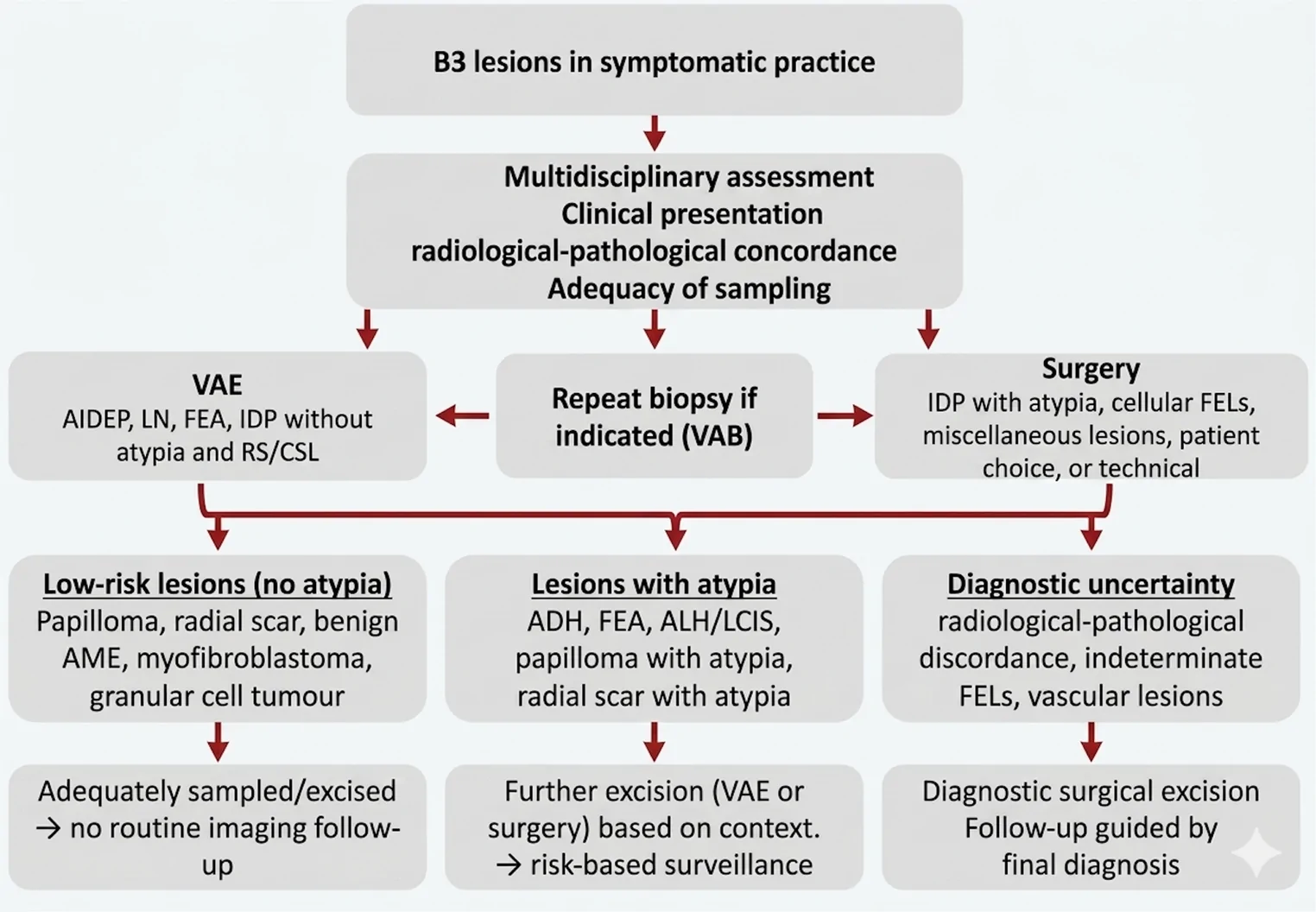
Management of B3 lesions in the symptomatic setting. Schematic overview of risk-adapted management following a B3 diagnosis on core needle biopsy. Initial assessment includes multidisciplinary review, clinical presentation, radiological-pathological concordance, and adequacy of sampling. Management may include repeat biopsy (typically vacuum-assisted biopsy [VAB]), vacuum-assisted excision (VAE) for selected lesions, or surgical excision where indicated. Subsequent pathways are stratified into low-risk lesions without epithelial atypia, lesions with epithelial atypia, and cases with diagnostic uncertainty or radiological–pathological discordance, which are typically managed by surgical excision. Follow-up is guided by the final diagnosis, with risk-based surveillance considered for atypical lesions

Based on current evidence, the management of symptomatic B3 lesions broadly parallels that of screen-detected lesions but should be tailored to the clinical presentation, with a lower threshold for intervention in cases of radiological-pathological discordance, mass-forming lesions, or persistent symptoms. VAE is the preferred method of further sampling and lesion assessment for many B3 lesions, including those without epithelial atypia and selected atypical lesions. Surgical excision may be performed if VAE is not technically feasible (e.g. very superficial lesions or those close to the nipple) or if preferred by the patient. Surgical excision is also recommended where there is radiological-pathological discordance (e.g. a palpable mass not accounted for by the results of the CNB/VAB) and for the evaluation of some specific B3 lesions diagnosed on CNB (Fig. 2). Pure epithelial atypia (e.g. AIDEP/ADH, LN, FEA) may be managed by VAE or surgical excision, according to the UK and international guidelines for B3 management summarised in Shaaban et al. 2025 [65].

Surgical excision is recommended for the definitive diagnosis of cellular fibroepithelial lesions (FELs) which account for a significant proportion of B3 lesions in younger patients. Recent guidelines consider enucleation an appropriate treatment when the differential diagnosis of a B3 cellular FEL on CNB is between cellular fibroadenoma and benign phyllodes tumour (PT). However, if borderline or malignant PT is suspected, surgical excision with clear margins is recommended [66, 67]. Notably, an increasing number of studies have reported favourable outcomes following VAE of small cellular fibroepithelial lesions that are not upgraded beyond benign PT [68].

In symptomatic practice, radial scars (≤ 1 cm) and complex sclerosing lesions (> 1 cm) are typically identified in association with clinical or imaging abnormalities [67]. Larger lesions may present as palpable masses, whereas smaller lesions are often incidental and not the direct cause of symptoms. Thorough sampling is essential, as larger lesions (e.g. >2 cm) may harbour unsampled malignancy despite the absence of epithelial atypia on CNB. Accordingly, further assessment by VAE or surgical excision, depending on lesion size, extent and technical considerations, is recommended. When the symptomatic target lesion has been completely removed by VAE or surgery, with radiological-pathological concordance, routine lesion-specific short-term imaging follow-up is generally unnecessary [23]. Clinical review is appropriate if symptoms recur. Where a concordant low-risk B3 lesion remains in situ (e.g. small RS/CSL without atypia), patients may be reassured and discharged or considered for limited interval imaging surveillance following MDT discussion. This approach also applies to patients with a diagnosis of papilloma without atypia on CNB. Surgical excision is the preferred method for evaluation of papillomas with epithelial atypia (AIDEP/ADH or LN). Diagnosis and management of papilloma with FEA remains uncertain [69, 70].

Screen-detected mucocele-like lesions (MLLs) without atypia typically carry a low risk of malignancy [71, 72]. In this context, imaging findings are often non-specific and variable; the most common presentation is incidental coarse heterogeneous calcifications on mammography. Occasionally, an asymmetry or mass may be detected with or without calcifications, and such lesions have a higher rate of upgrade to malignancy at excision [73]. When symptomatic MLLs present as a mass lesion, the absence of atypia on CNB may reflect sampling limitations, and VAE or surgical excision should be considered to exclude mucinous carcinoma [73].

Management of spindle cell lesions depends on the specific pathological diagnosis, as these represent a heterogeneous group. After exclusion of fibromatosis-like metaplastic breast carcinoma with application of a range of immunohistochemical markers, lesions should be managed individually with emphasis on diagnostic certainty rather than routine removal. Desmoid fibromatosis in the breast is a rare benign lesion typically presenting as a locally infiltrative symptomatic mass that can closely mimic malignancy clinically and radiologically [74–76]. Management of desmoid tumours has shifted from primary surgical resection to a risk-adapted approach prioritising observation. Modern consensus guidelines, including those from the National Comprehensive Cancer Network (NCCN) and the European Desmoid Tumour Working Group, recommend initial observation for stable disease and reserve surgery for refractory or rapidly progressive cases [77–80]. This evolution acknowledges the high recurrence rates associated with surgical excision and the potential for spontaneous stabilisation. Accordingly, once the diagnosis of fibromatosis is confidently established on CNB (supported by typical histology, β-catenin staining and molecular analysis for *CTNNB1* or *APC* gene mutation where available) and malignancy has been excluded, management should be individualised through a specialist soft tissue MDT, balancing symptoms, lesion behaviour/progression, and patient preference.

Adenomyoepithelioma (AME) may be difficult to diagnose on CNB, but even if diagnosed on such limited sampling, it should be surgically excised to confirm the diagnosis and exclude more serious pathology, which may rarely be seen in the form of atypical or malignant features in either glandular or myoepithelial cell components. As AME, including the classical form, is associated with an increased risk of recurrence [67], surgical excision should aim to achieve a clear margin.

Miscellaneous B3 lesions such as myofibroblastoma, nodular fasciitis and granular cell tumour may present as mass lesions and mimic malignancy but are rarely associated with epithelial atypia and have negligible metastatic potential. While many breast units would still consider surgical excision optimal management, taking account of patient preference, this may not always be necessary when the diagnosis is confidently established on CNB with appropriate ancillary studies, and the imaging and pathology findings are concordant. In contrast, solitary fibrous tumour represents a true neoplasm with inherent biological risk and complete surgical excision is required to mitigate the risk of recurrence or metastasis [81]. Pseudoangiomatous stromal hyperplasia (PASH) is typically classified as a benign lesion (B2). However, cellular PASH may present as a cellular spindle cell proliferation on CNB, leading to a B3 designation. If a confident diagnosis of cellular PASH is established, with radiological-pathological concordance, surgical excision is generally not required.

Where multiple B3 components co-exist on CNB, e.g. AIDEP/ADH associated with FEA or LN, or FELs with LCIS, published series indicate that the risk of upgrade and consequently management is dictated by the most aggressive component (Table 2). Decisions should be included in a managed multidisciplinary pathway, ensuring triple assessment and accounting for any discordance.

### Follow-up strategies in symptomatic practice

Routine lesion-specific surveillance is generally not required for patients with B3 lesions without epithelial atypia, regardless of whether the lesion is screen-detected or symptomatic, provided adequate diagnostic assessment or excision has been completed and radiological-pathological concordance achieved. In contrast, the role of imaging follow-up after the management of B3 lesions with epithelial atypia remains controversial. This reflects two distinct clinical considerations: the short-term risk of residual or unsampled atypical disease and the long-term increased risk of subsequent breast cancer associated with the underlying atypical lesion. Historically, routine surveillance has been mandated following VAB or VAE because complete removal of the lesion with epithelial atypia cannot always be guaranteed, leaving a risk of occult malignancy. Conversely, when complete surgical excision achieves clear margins and radiological–pathological concordance, the rationale for surveillance shifts from detecting localised residual disease to managing the patient’s elevated background risk of future breast cancer, which may occur in either breast.

Given that the increased breast cancer risk associated with atypical lesions persists for many years, surveillance limited to a defined short-term period may not adequately address this long-term risk. Nevertheless, the optimal duration, frequency, and modality of follow-up remain uncertain, and considerable variation exists among international guidelines. In 2018, the United Kingdom (UK) National Health Service Breast Screening Programme (NHSBSP) implemented mandatory annual mammographic surveillance for 5 years following the diagnosis of certain atypical lesions to generate prospective outcome data [16]. In contrast, other international frameworks adopt a more cautious approach. The European guidelines [10] recommend surveillance without specifying its frequency or duration, while the Third International Consensus [15] did not reach a vote on follow-up recommendations. The American Society of Breast Surgeons (ASBrS) [82] advises imaging surveillance at 6, 12, and 24 months following management, whereas NCCN [66] advocates a risk-adapted strategy incorporating annual mammography (with digital breast tomosynthesis where available) and supplemental breast MRI for women with an estimated lifetime breast cancer risk of ≥ 20%. Patients with confirmed ADH, LN, or mixed atypia should ideally be incorporated into established high-risk management pathways that include formal risk assessment, discussion of risk-reducing strategies, including chemoprevention, and risk-adapted imaging surveillance [7, 16, 23, 83]. Consequently, follow-up practices vary considerably between institutions. Some centres return patients to routine population-based screening after complete excision, whereas others advocate enhanced mammographic surveillance, particularly for younger women or those with additional risk factors.

Therefore, further evidence is required to determine whether follow-up strategies should be guided primarily by the adequacy and completeness of lesion removal or by the underlying biological risk associated with atypical breast lesions. Recent data from the Sloane screen-detected atypia audit demonstrated that the risk of cancer at years 1 and 3 was low, comparable to the general population, confirming that the VAE pathway is safe with negligible underestimation of malignancy [23, 24]. Based on these findings, the recent NHSBSP update has abolished the requirement for routine annual mammographic follow-up for screen-detected atypia, returning these patients directly to the standard triennial screening pool [25].

Crucially, there is little to no evidence regarding follow-up strategies for atypia diagnosed in the symptomatic setting. While many units empirically manage these patients similarly to those with screen-detected lesions, symptomatic cohorts often include younger women with dense breast tissue, which reduces the negative predictive value of conventional mammography. While supplemental ultrasound or MRI is often deployed case-by-case, surveillance methodologies for patients under the population screening age are not standardised. For women approaching screening age, a planned transition into the national screening programme is prudent; however, management protocols for much younger women remain challenging. Given these gaps in evidence, standard annual imaging follow-up is currently adopted in many centres for patients diagnosed with breast epithelial atypia presenting in the symptomatic practice while further data are accumulated. To address this, the National Coordinating Committee for Breast Pathology, in collaboration with the Association of Breast Surgery (ABS), is establishing a multicentre retrospective UK audit evaluating upgrade rates and long-term outcomes in symptomatic patients under 50 years of age with atypia. Data from this initiative will be pivotal in establishing evidence-based recommendations and refining surveillance techniques and strategies for this underrepresented cohort.

## Advanced imaging (MRI and CEM) in symptomatic b3 management

While MRI and CEM are superior for assessing disease extent in dense breasts, their specific role in symptomatic B3 lesions requires a nuanced approach [22, 84]. Proactive use of MRI or CEM following a symptomatic B3 diagnosis should be considered when there is significant radiological-pathological discordance or to exclude occult multicentricity that may alter the surgical plan. However, current evidence remains limited on whether a “negative” MRI can safely preclude surgical excision; a negative scan does not necessarily rule out the risk of upgrade associated with the original B3 focus. In this context, CEM is emerging as a pragmatic, cost-effective alternative to MRI, offering potentially comparable sensitivity for identifying associated malignancy [22, 84]. The role of functional imaging in B3 lesions remains under evaluation, and all cases should be discussed within an MDT setting.

### Limitations of current evidence

Evidence guiding the management of B3 lesions in symptomatic practice is limited. B3 lesions with epithelial atypia are uncommon in symptomatic practice, and data are derived predominantly from retrospective studies with small sample sizes, varied definitions, variable biopsy techniques, and selection bias. Most available data derive from screening cohorts, and many studies primarily report upgrade rates rather than providing robust evidence to inform surveillance and management strategies. An additional limitation relates to recognised interobserver variability in the histopathological diagnosis of several B3 entities. Studies have demonstrated substantial interobserver variability in the assessment of borderline lesions such as FEA, LN, particularly ALH, and FELs which may be under-recognised or underdiagnosed on CNB [38–40]. Such variability may influence reported upgrade rates and complicate comparisons across studies.

While guidance based on available evidence and expert consensus, such as presented in this review, remains valuable in this context, recommendations are necessarily subject to revision as new data emerge. Although most published data originate from the UK and European practice, these entities are universally recognised as high-risk lesions, and the underlying management principles remain broadly applicable across healthcare systems.

### Future directions

Priorities for future research include multi-centre data collation of B3 lesions from symptomatic patients, the development of prospective, ideally randomised, studies in this setting, and the standardisation of pathological reporting, ideally with centralised pathological review. From a biological perspective, improved understanding of the mechanisms underlying breast cancer development may enhance the management of B3 lesions, whether symptomatic or screen-detected, by enabling more accurate assessment of malignant transformation risk and identifying opportunities for early interception strategies. Artificial intelligence algorithms in radiology may be able in the future to refine the diagnostic criteria and improve targeting of suspicious lesions [85]. The identification and validation of reliable biomarkers, together with emerging genomic risk assessment approaches such as SNP-based risk profiling used in initiatives like MyPEBS [86], may further refine personalised risk stratification and guide tailored surveillance or intervention strategies. Therefore, the development of dedicated, evidence-based guidelines for the management and follow-up of symptomatic B3 lesions remains an urgent priority. In this light, there is an increasing tendency to de-escalate surgery in view of recognised overtreatment for some of these lesions.

## Conclusions

The management of B3 lesions in symptomatic practice remains challenging. Given the limited direct evidence, current management remains largely individualised, MDT-based and aligned to the screen detected lesions guidelines. Improvements in tailored, risk-adapted approaches that integrate pathological, radiological and clinical findings alongside patient preference are required. While VAE and surveillance are appropriate for many patients, surgical excision continues to be advised for selected symptomatic B3 lesions. Prospective studies and formal consensus exercises are required to establish structured recommendations and standardise evidence-based follow-up in symptomatic lesions with epithelial atypia.

## Supplementary information

Below is the link to the electronic supplementary material.

Supplementary Material 1 (DOCX 16.2 KB)

## Data Availability

Not applicable (as no raw data are generated).

## References

[1] Lee AHS, Carder P, Deb R (June2016) Guidelines for non-operative diagnostic procedures and reporting in breast cancer screening. The Royal College of Pathologists, London

[2] Rakha EA, Ellis IO (2007) An overview of assessment of prognostic and predictive factors in breast cancer needle core biopsy specimens. J Clin Pathol 60:1300–1306 10.1136/jcp.2006.045377 PMC2095575 17630399

[3] El-Sayed ME, Rakha EA, Reed J et al (2008) Audit of performance of needle core biopsy diagnoses of screen detected breast lesions. Eur J Cancer 44:2580–2586 10.1016/j.ejca.2008.05.024 18632261

[4] Rakha EA, Quinn C, Raymond W et al (2025) Classification of Fibroepithelial Lesions of the Breast in Core Needle Biopsy With Implications for Further Management. Mod Pathol 38:100734 10.1016/j.modpat.2025.100734 39947480

[5] Huang XC, Hu XH, Wang XR et al (2018) A comparison of diagnostic performance of vacuum-assisted biopsy and core needle biopsy for breast microcalcification: a systematic review and meta-analysis. Ir J Med Sci 187:999–1008 10.1007/s11845-018-1781-6 29549564

[6] Sharma N, Theis S, Vogelmann T et al (2026) Vacuum-assisted breast biopsy vs core needle biopsy: a systematic review and meta-analysis. Eur Radiol. 10.1007/s00330-025-12299-1 PMC13212802 41553473

[7] American Society of Breast Surgeons (2016) Consensus guideline on concordance assessment of image-guided breast biopsies and management of borderline or high-risk lesions. Available from: https://associationofbreastsurgery.org.uk/media/0naeglap/asbs-concordance_and_high-risk-lesions.pdf. Accessed Feb 2026

[8] Grabenstetter A (2025) High-Risk Breast Lesions on Core Biopsy: Rates of Upgrade and Management Recommendations. Surg Pathol Clin 18:707–723 10.1016/j.path.2025.05.001 PMC13537485 41224414

[9] Bahl M, Barzilay R, Yedidia AB et al (2018) High-Risk Breast Lesions: A Machine Learning Model to Predict Pathologic Upgrade and Reduce Unnecessary Surgical Excision. Radiology 286:810–818 10.1148/radiol.2017170549 29039725

[10] Rubio IT, Wyld L, Marotti L et al (2024) European guidelines for the diagnosis, treatment and follow-up of breast lesions with uncertain malignant potential (B3 lesions) developed jointly by EUSOMA, EUSOBI, ESP (BWG) and ESSO. Eur J Surg Oncol 50:107292 10.1016/j.ejso.2023.107292 38061151

[11] National Coordinating Group for Breast Screening Pathology (1997) Guidelines for cytology and histology in breast cancer screening, vol 49. NHSBSP Publication No

[12] Sloane JP, Amendoeira I, Apostolikas N et al (1999) Consistency achieved by 23 European pathologists from 12 countries in diagnosing breast disease and reporting prognostic features of carcinomas. European Commission Working Group on Breast Screening Pathology. Virchows Arch 434:3–10 10.1007/s004280050297 10071228

[13] Ellis IO, Humphreys S, Michell M et al (2004) Best Practice 179. Guidelines for breast needle core biopsy handling and reporting in breast screening assessment. J Clin Pathol 57:897–902 10.1136/jcp.2003.010983 PMC1770422 15333647

[14] Willers N, Neven P, Floris G et al (2023) The Upgrade Risk of B3 Lesions to (Pre)Invasive Breast Cancer After Diagnosis on Core Needle or Vacuum Assisted Biopsy. A Belgian National Cohort Study. Clin Breast Cancer 23:e273–e280 10.1016/j.clbc.2023.03.006 37085379

[15] Elfgen C, Leo C, Kubik-Huch RA et al (2023) Third International Consensus Conference on lesions of uncertain malignant potential in the breast (B3 lesions). Virchows Arch 483:5–20 10.1007/s00428-023-03566-x PMC10326140 37330436

[16] Pinder SE, Shaaban A, Deb R et al (2018) NHS Breast Screening multidisciplinary working group guidelines for the diagnosis and management of breast lesions of uncertain malignant potential on core biopsy (B3 lesions). Clin Radiol 73:682–692 10.1016/j.crad.2018.04.004 29773220

[17] Guirguis MS, Yang WT (2023) Overview of incidental breast lesions. Br J Radiol 96:20211326 10.1259/bjr.20211326 PMC9975524 35451861

[18] Cullinane C, Byrne J, Kelly L et al (2022) The positive predictive value of vacuum assisted biopsy (VAB) in predicting final histological diagnosis for breast lesions of uncertain malignancy (B3 lesions): a systematic review & meta-analysis. Eur J Surg Oncol 48:1464–1474 10.1016/j.ejso.2022.04.005 35491362

[19] Dalton JC, Nierenberg TC, Leonard A et al (2025) Risk prediction models for malignancy upgrade in high-risk breast lesions: a qualitative systematic review. Breast Cancer Res Treat 215:3 10.1007/s10549-025-07834-z 41269377

[20] Cohen M, Benhaim S, Coudray AJ et al (2020) When to do surgical resection for atypical breast lesions: results of a prospective cohort of 518 lesions. Surg Oncol 34:80–85 10.1016/j.suronc.2020.04.002 32891358

[21] Lord SJ, Lei W, Craft P et al (2007) A systematic review of the effectiveness of magnetic resonance imaging (MRI) as an addition to mammography and ultrasound in screening young women at high risk of breast cancer. Eur J Cancer 43:1905–1917 10.1016/j.ejca.2007.06.007 17681781

[22] Ermans SJE, Maas R, Hill TM et al (2026) Comparison of contrast-enhanced mammography and breast MRI in tumour extent assessment: a systematic review and meta-analysis. Eur J Surg Oncol 52:111410 10.1016/j.ejso.2026.111410 41576518

[23] Freeman K, Jenkinson D, Clements K et al (2024) Atypia detected during breast screening and subsequent development of cancer: observational analysis of the Sloane atypia prospective cohort in England. BMJ 384:e077039 10.1136/bmj-2023-077039 PMC10831586 38302129

[24] Freeman K, Mansbridge A, Stobart H et al (2024) Evidence-informed recommendations on managing breast screening atypia: perspectives from an expert panel consensus meeting reviewing results from the Sloane atypia project. Br J Radiol 97:324–330 10.1093/bjr/tqad053 38265306

[25] NHS England (2010) Clinical guidance for breast cancer screening assessment [Internet]. London: GOV.UK [updated 2026; cited 2026 May 20]. Available from: https://www.gov.uk/government/publications/breast-screening-clinical-guidelines-for-screening-management/clinical-guidance-for-breast-cancer-screening-assessment

[26] Preibsch H, Wanner LK, Staebler A et al (2018) Malignancy rates of B3-lesions in breast magnetic resonance imaging - do all lesions have to be excised? BMC Med Imaging 18:27 10.1186/s12880-018-0271-7 PMC6131767 30200900

[27] Maclean GM, Courtney SP, Umeh H et al (2013) Is mode of presentation of B3 breast core biopsies (screen-detected or symptomatic) a distinguishing factor in the final histopathologic result or risk of diagnosis of malignancy? World J Surg 37:2607–2612 10.1007/s00268-013-2191-6 23963348

[28] Sheikh SE, Rathbone M, Chaudhary K et al (2022) Rates and Outcomes of Breast Lesions of Uncertain Malignant Potential (B3) benchmarked against the National Breast Screening Pathology Audit; Improving Performance in a High Volume Screening Unit. Clin Breast Cancer 22:381–390 10.1016/j.clbc.2022.02.004 35260351

[29] London SJ, Connolly JL, Schnitt SJ et al (1992) A prospective study of benign breast disease and the risk of breast cancer. JAMA 267:941–944 1734106

[30] Hartmann LC, Sellers TA, Frost MH et al (2005) Benign breast disease and the risk of breast cancer. N Engl J Med 353:229–237 10.1056/NEJMoa044383 16034008

[31] Catanzariti F, Avendano D, Cicero G et al (2021) High-risk lesions of the breast: concurrent diagnostic tools and management recommendations. Insights Imaging 12:63 10.1186/s13244-021-01005-6 PMC8155169 34037876

[32] Lucioni M, Rossi C, Lomoro P et al (2021) Positive predictive value for malignancy of uncertain malignant potential (B3) breast lesions diagnosed on vacuum-assisted biopsy (VAB): is surgical excision still recommended? Eur Radiol 31:920–927 10.1007/s00330-020-07161-5 32816199

[33] Kocjan G, Bourgain C, Fassina A et al (2008) The role of breast FNAC in diagnosis and clinical management: a survey of current practice. Cytopathology 19:271–278 10.1111/j.1365-2303.2008.00610.x 18821945

[34] Schilling K, Love N (1995) Screening and diagnostic breast imaging procedures. A look at lesions through a radiologist’s eyes. Postgrad Med 98:44 – 6, 51, 56 passim 7567723

[35] Starikov A, Askin G, Blackburn A et al (2021) Mode of detection matters: Differences in screen-detected versus symptomatic breast cancers. Clin Imaging 80:11–15 10.1016/j.clinimag.2021.06.032 34218078

[36] NHS England (2020) The surveillance of women at very high risk of developing breast cancer [Internet]. London: GOV.UK [updated 2025; cited 2026 May 20]. Available from: https://www.gov.uk/government/publications/breast-screening-higher-risk-women-surveillance-protocols/the-surveillance-of-women-at-very-high-risk-of-developing-breast-cancer

[37] NHS England (2020) Eligibility criteria and screening protocols for women at very high risk of breast cancer [Internet]. London: GOV.UK [updated 2025; cited 2026 May 20]. Available from: https://www.gov.uk/government/publications/breast-screening-higher-risk-women-surveillance-protocols/eligibility-criteria-and-screening-protocols-for-women-at-very-high-risk-of-breast-cancer

[38] El Sheikh S, Mansour MA, Provenzano E et al (2025) Diagnostic interobserver variability of atypia assessment in columnar cell lesions among a group of expert breast pathologists in the United Kingdom and the Republic of Ireland, on behalf of the UK national coordinating committee for breast pathology. Histopathology 86:953–966 10.1111/his.15402 PMC11964577 39704183

[39] Dessauvagie BF, Lee AHS, Meehan K et al (2018) Interobserver variation in the diagnosis of fibroepithelial lesions of the breast: a multicentre audit by digital pathology. J Clin Pathol 71:672–679 10.1136/jclinpath-2017-204977 29440134

[40] Gomes DS, Porto SS, Balabram D et al (2014) Inter-observer variability between general pathologists and a specialist in breast pathology in the diagnosis of lobular neoplasia, columnar cell lesions, atypical ductal hyperplasia and ductal carcinoma in situ of the breast. Diagn Pathol 9:121 10.1186/1746-1596-9-121 PMC4071798 24948027

[41] Shin HJ, Kim HH, Kim SM et al (2008) Screening-detected and symptomatic ductal carcinoma in situ: differences in the sonographic and pathologic features. AJR Am J Roentgenol 190:516–525 10.2214/AJR.07.2206 18212241

[42] Barnes NL, Dimopoulos N, Williams KE et al (2014) The frequency of presentation and clinico-pathological characteristics of symptomatic versus screen detected ductal carcinoma in situ of the breast. Eur J Surg Oncol 40:249–254 10.1016/j.ejso.2013.12.013 24433818

[43] Venkatesh SL, Oseni TO, Bahl M (2021) Symptomatic ductal carcinoma in situ (DCIS): Upstaging risk and predictors. Clin Imaging 73:101–107 10.1016/j.clinimag.2020.11.050 33360004

[44] Kim J, Lee S, Bae S et al (2012) Comparison between screen-detected and symptomatic breast cancers according to molecular subtypes. Breast Cancer Res Treat 131:527–540 10.1007/s10549-011-1836-0 22042364

[45] Corradini AG, Cremonini A, Cattani MG et al (2021) Which type of cancer is detected in breast screening programs? Review of the literature with focus on the most frequent histological features. Pathologica 113:85–94 10.32074/1591-951X-123 PMC8167395 34042090

[46] Lau SS, Cheung PS, Wong TT et al (2016) Comparison of clinical and pathological characteristics between screen-detected and self-detected breast cancers: a Hong Kong study. Hong Kong Med J 22:202–209 10.12809/hkmj154575 27022189

[47] Rageth CJ, Rubenov R, Bronz C et al (2019) Atypical ductal hyperplasia and the risk of underestimation: tissue sampling method, multifocality, and associated calcification significantly influence the diagnostic upgrade rate based on subsequent surgical specimens. Breast Cancer 26:452–458 10.1007/s12282-018-00943-2 PMC6570781 30591993

[48] Baker J, Noguchi N, Marinovich ML et al (2024) Atypical ductal or lobular hyperplasia, lobular carcinoma in-situ, flat epithelial atypia, and future risk of developing breast cancer: Systematic review and meta-analysis. Breast 78:103807 10.1016/j.breast.2024.103807 PMC11415589 39270543

[49] Rudin AV, Hoskin TL, Fahy A et al (2017) Flat Epithelial Atypia on Core Biopsy and Upgrade to Cancer: a Systematic Review and Meta-Analysis. Ann Surg Oncol 24:3549–3558 10.1245/s10434-017-6059-0 28831724

[50] Nakhlis F, Baker GM, Li T et al (2025) Incidence of Adjacent Synchronous Ipsilateral Infiltrating Carcinoma and/or Ductal Carcinoma In Situ in Patients Diagnosed with Flat Epithelial Atypia by Core Needle Biopsy (TBCRC 034). Ann Surg Oncol 32:2578–2584 10.1245/s10434-024-16762-z 39751983

[51] Verschuur-Maes AH, van Deurzen CH, Monninkhof EM et al (2012) Columnar cell lesions on breast needle biopsies: is surgical excision necessary? A systematic review. Ann Surg 255:259–265 10.1097/SLA.0b013e318233523f 21989373

[52] Uzan C, Mazouni C, Rossoni C et al (2021) Prospective Multicenter Study Validate a Prediction Model for Surgery Uptake Among Women with Atypical Breast Lesions. Ann Surg Oncol 28:2138–2145 10.1245/s10434-020-09107-z 32920723

[53] Verheyden C, Pages-Bouic E, Balleyguier C et al (2016) Underestimation Rate at MR Imaging-guided Vacuum-assisted Breast Biopsy: A Multi-Institutional Retrospective Study of 1509 Breast Biopsies. Radiology 281:708–719 10.1148/radiol.2016151947 27355898

[54] Speer ME, Huang ML, Dogan BE et al (2018) High risk breast lesions identified on MRI-guided vacuum-assisted needle biopsy: outcome of surgical excision and imaging follow-up. Br J Radiol 91:20180300 10.1259/bjr.20180300 PMC6350470 29947265

[55] Santucci D, Faiella E, Calabrese A et al (2019) Our Radiological Experience on B3 Lesions: Correlation Between Mammographic and MRI Findings With Histologic Definitive Result. Clin Breast Cancer 19:e643–e653 10.1016/j.clbc.2019.06.004 31377083

[56] Llewellyn A, Brown A, Morel J et al (2026) To VAE or not to VAE: outcomes of radial scars/complex sclerosing lesions and papillary lesions without atypia in the King’s College Hospital breast service (2017–2023). Histopathology 88:583–590 10.1111/his.70017 PMC12793823 41037018

[57] Okamoto S, Chen ST, Covelli JD et al (2020) High-risk lesions diagnosed at MRI-guided vacuum-assisted breast biopsy: imaging characteristics, outcome of surgical excision or imaging follow-up. Breast Cancer 27:405–414 10.1007/s12282-019-01032-8 31838725

[58] Hartmann LC, Degnim AC, Santen RJ et al (2015) Atypical hyperplasia of the breast–risk assessment and management options. N Engl J Med 372:78–89 10.1056/NEJMsr1407164 PMC4347900 25551530

[59] Renshaw AA, Gould EW (2016) Long term clinical follow-up of atypical ductal hyperplasia and lobular carcinoma in situ in breast core needle biopsies. Pathology 48:25–29 10.1016/j.pathol.2015.11.015 27020205

[60] Sherman ME, Vierkant RA, Winham SJ et al (2024) Benign Breast Disease and Breast Cancer Risk in the Percutaneous Biopsy Era. JAMA Surg 159:193–201 10.1001/jamasurg.2023.6382 PMC10719829 38091020

[61] Dyrstad SW, Yan Y, Fowler AM et al (2015) Breast cancer risk associated with benign breast disease: systematic review and meta-analysis. Breast Cancer Res Treat 149:569–575 10.1007/s10549-014-3254-6 25636589

[62] McEvoy MP, Coopey SB, Mazzola E et al (2015) Breast Cancer Risk and Follow-up Recommendations for Young Women Diagnosed with Atypical Hyperplasia and Lobular Carcinoma In Situ (LCIS). Ann Surg Oncol 22:3346–3349 10.1245/s10434-015-4747-1 26242364

[63] Sergesketter AR, Thomas SM, Fayanju OM et al (2019) The Influence of Age on the Histopathology and Prognosis of Atypical Breast Lesions. J Surg Res 241:188–198 10.1016/j.jss.2019.03.047 PMC6571073 31028940

[64] Nierenberg TC, Dalton JC, Howell R et al (2025) Risk assessment tools for women with breast atypia: a systematic review. Am J Surg 250:116598 10.1016/j.amjsurg.2025.116598 PMC12441980 40925271

[65] Shaaban AM, McMahon MA, Sharma N (2025) Recent advances in the diagnosis and management of B3 lesions. Diagn Histopathology 31:145–151

[66] Gradishar WJ, Moran MS, Abraham J et al (2026) NCCN clinical practice guidelines in oncology. Breast Cancer v1. (Downloaded from NCCN.org on 08 Feb 2026)

[67] WHO Classification of Tumours Editorial Board (2026) Breast Tumours, Beta version; 6th edition. International Agency for Research on Cancer, Lyon (France)

[68] Gil M, Coelho BA, Couto HL et al (2024) Vacuum-assisted excision: a safe minimally invasive option for benign phyllodes tumor diagnosis and treatment-a systematic review and meta-analysis. Front Oncol 14:1394116 10.3389/fonc.2024.1394116 PMC11130386 38807769

[69] Rakha EA (2026) Contextual interpretation of luminal phenotype in low-grade breast epithelial proliferations. J Clin Pathol. 10.1136/jcp-2026-210821 42215291

[70] Rakha EA, Tan PH, Raymond WA (2026) The spectrum of breast in situ papillary carcinomas with invasion and invasive breast carcinomas with papillary features: an overview of histological subtypes and diagnostic challenges. Histopathology 88:747–768 10.1111/his.70072 PMC12891923 41404713

[71] Zhang G, Ataya D (2018) Mucocele-like lesions diagnosed on breast core biopsy: Low risk of upgrade and subsequent carcinoma. Breast J 24:314–318 10.1111/tbj.12929 29024198

[72] Rakha EA, Shaaban AM, Haider SA et al (2013) Outcome of pure mucocele-like lesions diagnosed on breast core biopsy. Histopathology 62:894–898 10.1111/his.12081 23402386

[73] Chandora A, Kahn AG, Zamora K (2024) Mucocele-like Lesions: Radiologic-Pathologic Correlation. J Breast Imaging 6:175–182 10.1093/jbi/wbae006 38394371

[74] Plaza MJ, Yepes M (2012) Breast fibromatosis response to tamoxifen: dynamic MRI findings and review of the current treatment options. J Radiol Case Rep 6:16–23 10.3941/jrcr.v6i3.897 PMC3370708 22690287

[75] Lacka DE, Nasierowska-Guttmejer A (2019) Fibromatosis - immunohistochemical evaluation, differential diagnosis from gastrointestinal tumors, and other mesenchymal tumours. Prz Gastroenterol 14:79–85 10.5114/pg.2019.83429 PMC6444105 30944681

[76] Lee YS, Joo MW, Shin SH et al (2024) Current treatment concepts for extra-abdominal desmoid-type fibromatosis: a narrative review. Cancers (Basel). 10.3390/cancers16020273 PMC10813957 38254764

[77] Desmoid Tumor Working G (2020) The management of desmoid tumours: a joint global consensus-based guideline approach for adult and paediatric patients. Eur J Cancer 127:96–107 10.1016/j.ejca.2019.11.013 32004793

[78] von Mehren M, Kane JM, Agulnik M et al (2022) Soft Tissue Sarcoma, Version 2.2022, NCCN Clinical Practice Guidelines in Oncology. J Natl Compr Canc Netw 20:815–833 10.6004/jnccn.2022.0035 PMC10186762 35830886

[79] Wu M, Hughes TM, Edirimanne S et al (2024) Breast Desmoid Tumours: A Review of the Literature. Breast J 2024:5803290 10.1155/2024/5803290 PMC11259505 39742373

[80] Kangas-Dick A, Ali M, Poss M et al (2024) Diagnosis and Management of Desmoid Fibromatosis of the Breast. World J Oncol 15:394–404 10.14740/wjon1844 PMC11092408 38751692

[81] Demicco EG, Park MS, Araujo DM et al (2012) Solitary fibrous tumor: a clinicopathological study of 110 cases and proposed risk assessment model. Mod Pathol 25:1298–1306 10.1038/modpathol.2012.83 22575866

[82] The American Society of Breast Surgeons (ASBrS) (2025) Resource Guide: Surgical Management of Benign or High-Risk Breast Lesions. https://www.breastsurgeons.org/docs/statements/asbrs-high-risk-lesions.pdf

[83] Bychkovsky B, Laws A, Katlin F et al (2022) Initiation and tolerance of chemoprevention among women with high-risk breast lesions: the potential of low-dose tamoxifen. Breast Cancer Res Treat 193:417–427 10.1007/s10549-022-06577-5 PMC8978759 35378642

[84] Widmayer P, Ferrari-Kanzler E, Haider SP et al (2026) A systematic comparison of abbreviated and unenhanced breast MRI protocols in women with mammographically dense breasts compared to full multiparametric breast MR imaging. Eur J Radiol 200:112825 10.1016/j.ejrad.2026.112825 41967277

[85] D’Archi S, Carnassale B, Sanchez AM et al (2025) Navigating the uncertainty of B3 breast lesions: diagnostic challenges and evolving management strategies. J Pers Med 15 10.3390/jpm15010036 PMC11766555 39852228

[86] Allweis TM, Hermann N, Berenstein-Molho R et al (2021) Personalized Screening for Breast Cancer: Rationale, Present Practices, and Future Directions. Ann Surg Oncol 28:4306–4317 10.1245/s10434-020-09426-1 33398646

