# Supplementary material for: Management of high-risk (B3) breast lesions in symptomatic practice: evidence review and practical recommendations for multidisciplinary care
Source: Virchows Arch. 2026 Aug 10;489(4):783–96. doi: 10.1007/s00428-026-04672-2 (PMC13714129; doi:10.1007/s00428-026-04672-2)
Supplement: Supplementary file 1 [file 428_2026_4672_MOESM1_ESM.docx]

**Supplementary Table 1.** Comparison of core needle biopsy, vacuum-assisted biopsy, vacuum-assisted excision, and surgical excision in breast practice

| **Technique** | **Main purpose** | **Typical needle/device** | **Tissue yield / extent of removal** | **Key strengths** | **Key limitations** | **Suitable lesion size / role** | **When surgery is preferred** | **Contraindications / cautions** |
| --- | --- | --- | --- | --- | --- | --- | --- | --- |
| **Core needle biopsy (CNB)** | First-line diagnostic biopsy for most solid lesions | 14G automated core needle | Small tissue cores; sampling only (not excision) | Widely available; quick; cost-effective; reliable for many masses | Sampling error; underestimation of DCIS/invasion; limited for calcifications | Most lesions requiring initial diagnosis | Discordant findings; insufficient sampling; confirmed malignancy requiring excision | Small sample size; lesion heterogeneity; difficult targeting |
| **Vacuum-assisted biopsy (VAB)** | Diagnostic biopsy requiring larger tissue volume (e.g. microcalcifications, B3 lesions) | 8G–11G vacuum-assisted device | Larger volume than CNB (≈3× tissue versus 14G CNB) | Improved sampling accuracy; lower underestimation; useful for calcifications | Still primarily sampling; limited margin assessment | Lesions where CNB is insufficient or equivocal | Malignancy requiring therapeutic excision; persistent discordance | Technical difficulty; bleeding risk; poor lesion visibility |
| **Vacuum-assisted excision (VAE)** | Percutaneous excision of selected benign and B3 lesions | 7G–10G vacuum-assisted device (larger gauges preferred) | Near complete or complete lesion removal (no formal margins) | Minimally invasive; avoids surgery in selected cases; diagnostic + therapeutic | No formal margin assessment: completeness decreases with size | Best for small lesions (commonly ≤15–30 mm); selected cases up to ~3 cm; larger lesions less reliable | Lesions >3 cm; suspected phyllodes; confirmed malignancy; incomplete excision; need for margins | Large size (relative ≥3 cm); proximity to skin/nipple; poor visibility; high mobility; bleeding risk |
| **Surgical excision** | Definitive diagnostic and therapeutic excision | Open surgical procedure | Complete lesion removal with margins | Gold standard; full histology; margin assessment | Invasive; requires theatre/anaesthesia; higher cost | All lesions requiring definitive treatment or margin assessment | Standard for malignancy, large lesions, phyllodes, discordant or incompletely excised lesions | Surgical/anaesthetic risk; patient comorbidity |

* DCIS: Ductal carcinoma in situ, G (gauge): Needle size.

* VAE size thresholds are not absolute and depend on lesion type, location, and operator experience. In practice, small lesions (≤15–30 mm) are most suitable for complete percutaneous excision. Lesions >3 cm show reduced likelihood of complete removal, and ≥5 cm is often considered a relative contraindication. Surgical excision remains the standard where margin assessment or oncological treatment is required.
